# Basketball Teams as Strategic Networks

**DOI:** 10.1371/journal.pone.0047445

**Published:** 2012-11-06

**Authors:** Jennifer H. Fewell, Dieter Armbruster, John Ingraham, Alexander Petersen, James S. Waters

**Affiliations:** 1 School of Life Sciences, Arizona State University, Tempe, Arizona, United States of America; 2 School of Mathematical and Statistical Sciences, Arizona State University, Tempe, Arizona, United States of America; 3 Center for Social Dynamics and Complexity, Arizona State University, Tempe, Arizona, United States of America; Technical University of Madrid, Italy

## Abstract

We asked how team dynamics can be captured in relation to function by considering games in the first round of the NBA 2010 play-offs as networks. Defining players as nodes and ball movements as links, we analyzed the network properties of degree centrality, clustering, entropy and flow centrality across teams and positions, to characterize the game from a network perspective and to determine whether we can assess differences in team offensive strategy by their network properties. The compiled network structure across teams reflected a fundamental attribute of basketball strategy. They primarily showed a centralized ball distribution pattern with the point guard in a leadership role. However, individual play-off teams showed variation in their relative involvement of other players/positions in ball distribution, reflected quantitatively by differences in clustering and degree centrality. We also characterized two potential alternate offensive strategies by associated variation in network structure: (1) whether teams consistently moved the ball towards their shooting specialists, measured as “uphill/downhill” flux, and (2) whether they distributed the ball in a way that reduced predictability, measured as team entropy. These network metrics quantified different aspects of team strategy, with no single metric wholly predictive of success. However, in the context of the 2010 play-offs, the values of clustering (connectedness across players) and network entropy (unpredictability of ball movement) had the most consistent association with team advancement. Our analyses demonstrate the utility of network approaches in quantifying team strategy and show that testable hypotheses can be evaluated using this approach. These analyses also highlight the richness of basketball networks as a dataset for exploring the relationships between network structure and dynamics with team organization and effectiveness.

## Introduction

Capturing the interactions among individuals within a group is a central goal of network analyses. Useful depictions of network structure should provide information about the networks purpose and functionality. But how do network attributes relate to functional outcomes at the group and/or individual levels? A useful context to ask this question is within small team networks. Teams occur everywhere across the broad array of biological societies, from cooperatively hunting carnivores to social insects retrieving prey [1]–[4], and are ubiquitous in human organizations. We define teams as groups of individuals working collaboratively and in a coordinated manner towards a common goal be it winning a game, increasing productivity, or increasing a common good [5]. Within teams, individuals must coordinate across different roles or tasks, with their performance outcomes being interdependent [4]–[6]. The success of the team is rarely a simple summation of the tools each individual brings. Instead it must emerge from the dynamic interactions of the group as a whole [7].

How can we capture the relevance of these interactions to team function? Because teams are dynamic systems, it makes sense to use network analyses to approach this problem. The game of basketball is based on a series of interactions, involving a tension between specialization and flexibility; players must work together to move the ball into the basket while anticipating and responding to the opposing team. Thus, plays that begin as set strategies evolve quickly into dynamic interactions [8]. Unlike many sports, the game does not revolve around a series of dyadic interactions (eg tennis, baseball) or a summation of individual efforts (track and field); it is dependent on a connected team network [9].

The dynamic between within-group cooperation and conflict, and group versus individual success, is an inherent feature of both human and biological social systems. This tension, exemplified in the distribution of shooting opportunities in a game across players, or by salary dispersion inequities in a team or organization, is a fundamental issue across cooperative systems [6], [10], [11]. The dynamic between specialization and flexibility also appears across systems. In prides of lions, for example, different females assume the roles of driving or flanking prey [1]. However, in both contexts individuals must flexibly change positions in a rapidly changing game. Finally, like almost all cohesive groups, teams must compete with other teams, and their success/failure is shaped by their ability to respond to those challenges. Unlike a lion pride or business organization, however, the success and failure of specific network interactions for a basketball team can be easily measured iteratively and in real time, as the team scores points or loses the ball to a superior defense.

To evaluate basketball teams as networks, we examined the offensive ball sequences by National Basketball Association (NBA) teams during the first round of the 2010 playoffs. We graphed player positions and inbound/outcomes as nodes, and ball movement among nodes (including shots to the basket) as edges. From the iterated offensive 24 second clocks, we recorded sequences of ball movement of each of the 16 play-off teams across two games. We used the compiled data to first ask whether we can capture the game of basketball through a transition network representing the mean flow of the ball through these sequences of play (a stochastic matrix), and secondly whether individual teams have specific network signatures. We then examined how different network metrics may be associated with variation in actual play strategy. We asked whether teams vary strategically in centrality of ball distribution, such that some teams rely more heavily on a key player, such as the point guard, to make decisions on ball movement. We used degree centrality to compare teams using this strategy with those in which the ball is distributed more evenly. We similarly used clustering analyses to examine relative connectedness among players within teams and to ask whether teams differentially engaged players across multiple positions. We also asked whether ball movement rate, measured as path length and path flow rate, could capture the perceived dichotomy of teams using dominant large players, usually centers, versus small ball teams that move the ball quickly across multiple players [12].

We were interested in whether network metrics can usefully quantify team decisions about how to most effectively coordinate players. We examined two network metrics that we hypothesized might capture different offensive strategies. One is to move the ball in a way that is unpredictable and thus less defensible. To measure network unpredictability we calculated team entropy, applying Shannons entropy to the transition networks as a proxy for the unpredictability of individual passing behavior among team players. Another, not mutually exclusive, strategy is to capitalize on individual expertise by moving the ball towards players with high probability of shooting success. In a sense, this strategy reflects a coordinated division of labor between ball distributors early in the play, transitioning to shooting specialists. We looked for evidence of this strategy using a metric of uphill/downhill flux, which estimates the average change in potential shooting percentage as the ball moves between players in relation to their differential percent shooting success. Uphill/downhill and team entropy both recognize the need for coordination within a team, but they emphasize different aspects of network dynamics; one capitalizes on individual specialization while the other emphasizes team cohesion.

## Methods

We recorded and analyzed transition networks for the 16 teams in televised games of the 2010 NBA first round play-offs. The sequential ball movement for each teams offensive plays was recorded across two games for each pair; games were picked haphazardly a priori, not based on outcome (analyzed games and outcomes in Table 1). For analysis, the five starting players for each team were assigned position numbers from 1–5, in the order of: (1) Point Guard; (2) Shooting Guard; (3) Small Forward; (4) Power Forward; (5) Center. All offensive plays with at least three of the five starters on the floor were included (player list in Table S1. This allowed us to equate positions with specific players within each team and to use player positions as nodes. Preliminary analyses indicated that offensive play paths were fairly consistent between the two games analyzed for the majority of teams, so sequences were pooled.

**Table 1:** Analyzed games and outcomes.

Matchup	Games	Game Winner	Series Winner
Bobcats vs. Magic	Game 1	Magic	Magic
Bobcats vs. Magic	Game 2	Magic	Magic
Cavaliers vs. Bulls	Game 2	Cavaliers	Cavaliers
Cavaliers vs. Bulls	Game 4	Cavaliers	Cavaliers
Hawks vs. Bucks	Game 3	Bucks	Hawks
Hawks vs. Bucks	Game 4	Bucks	Hawks
Celtics vs. Heat	Game 1	Celtics	Celtics
Celtics vs. Heat	Game 3	Celtics	Celtics
Lakers vs. Thunder	Game 1	Lakers	Lakers
Lakers vs. Thunder	Game 2	Lakers	Lakers
Jazz vs. Nuggets	Game 1	Nuggets	Jazz
Jazz vs. Nuggets	Game 4	Jazz	Jazz
Mavericks vs. Spurs	Game 5	Mavericks	Spurs
Mavericks vs. Spurs	Game 6	Spurs	Spurs
Suns vs. Blazers	Game 1	Blazers	Suns
Suns vs. Blazers	Game 6	Suns	Suns

For initial analyses, all possible start-of-play (inbounds, rebounds and steals) and outcomes (successful/failed two point or three point shots, fouls, shooting fouls with different success outcomes, steals and turnovers) were recorded as nodes. Data per offensive play generated a sequential pathway [9], [13]. The cumulative paths throughout the game were combined to generate a weighted graph of ball movement with possession origin, players and possession outcomes as nodes and ball movement between those nodes as directed edges.

Although we chose games haphazardly, the differential in total points in analyzed games generally reflected outcomes for the play-off round (Table 1). The primary exception was the two Atlanta Hawks/Milwaukee Bucks games, in which the Bucks beat the Hawks in the series, but were defeated by a mean of 12.5 points during the two focal games. In the analyzed Dallas Mavericks/San Antonio Spurs games, Dallas won by a mean differential of 6 points, but the Spurs beat the Mavericks in the play-off series by a mean differential of 0.5; wins were split across the two games analyzed (Games 5 and 6).

### Network Analyses

We generated weighted graphs from the cumulative transition probabilities. When all data were analyzed, almost all nodes became connected, making it difficult to differentiate across graphs. Therefore, we generated a series of weighted graphs at increasing cut-off weights from the 30th to 70th percentiles (with the 30th percentile graphs highlighting only the most frequently seen transitions). This allowed us to analyze changes in network structure as we move from the most likely links between players to those that were least frequent. We used the entire matrix of transitions for each team to perform structural network analyses [12], [14], adapted for offensive plays in a basketball game. Metrics included: path length, path flow rate, degree centrality, clustering coefficient, individual and team entropy, individual and team flow centrality, shooting efficiency flux.

Path length and path flow rate compared the number of passes and the speed of ball movement involved in team play. Path length simply included the number of passes between players per play, ignoring inbound and outcome nodes. Paths included all between-player edges, such that a given player could be involved twice or more across the path. Path flow rate was calculated as the number of edges per unit time from inbound to shot clock time at the end of the play. To calculate degree centrality we used the weighted graphs from iterated offensive plays across the two games. However, we aggregated outcome data into two categories of shoot and other, to reduce weighting bias from multiple outcome nodes. Degree was first calculated per position as the weighted sum of total out-edges per player. The relative distributions of player degrees were then calculated across the graph, such that a homogeneous graph (connectivity distributed most equally across all players) has zero degree centrality. For a weighted graph 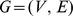 with weights summing to 1 and a vertex of maximal degree 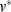 the degree centrality is then:
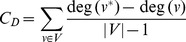
(1)


To calculate team entropy, we first determined individual player entropy. For this metric we excluded inbound passes because of the strong weight of the inbound edge. We included outcome, because the possibility of shooting the ball represents a decision point contributing to uncertainty of ball movement. As with centrality, outcomes were collapsed into two node categories of shooting or not shooting. We used Shannons entropy [15], 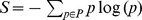, to measure the uncertainty of ball transitions between any player or outcome.

We then combined player entropies to determine entropy of the whole team. There are multiple ways to calculate network entropy. One possibility is to use a simple averaging of player entropies. A second is Markov chain entropy, which incorporates the conditional probability of any given player moving the ball to any other player, conditioned on the probability that the given player has the ball. However, from the opposing teams perspective, the real uncertainty of team play is the multiplicity of options across all ball movements rather than just across players. We thus calculated a whole-network or Team Entropy from the transition matrix describing ball movement probabilities across the five players and the two outcome options.

We used individual flow centrality to characterize player/position importance within the ball distribution network [16]. Individual player flow centrality was calculated as the number of passing sequences across all plays in which they were one of the nodes, normalized by the total number of plays. We also calculated a more restricted flow centrality that included only player appearances as one of the last three nodes before an outcome. This allowed us to focus on the set-up phase for a scoring drive and the actual scoring attempt. We compared this more restricted flow centrality for successful versus unsuccessful plays; this success/failure ratio was considered as a measure of the utility of an individual player to team success.

To capture a teams ability to move the ball towards their better shooters, we developed a metric we call uphill/downhill flux, defined as the average change in potential shooting percentage per pass. A team that has a high positive uphill/downhill flux moves the ball consistently to their better shooters; a team that with a negative value moves the ball on average to the weaker shooters. The latter can happen if the ball distributor (e.g. the Point Guard) is also the best shooter on the team. Letting 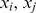 be the shooting percentages for players 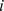 and 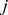 and 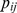 the probability of a pass from player 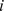 to player 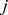, we define the uphill/downhill flux as:
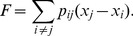
(2)


Finally, we wanted to compare teams in terms of relative player involvement, such that we can differentiate those teams for which most players are interconnected from those that rely consistently on a defined subset for offensive plays. One way to do so is to look for the occurrence of triangles, or connected 3-node subgraphs within the network. Teams with higher connectedness will contain more cases in which sets of 3 players have a link to each other; the maximum number of these triangles in a group of 5 players is 10. The clustering coefficient measures the number of triangles in a network as a percentage of all possible triangles. However, a single evaluation of this metric is again problematic. If we use all ball movement data, all nodes become connected to all other nodes, and the clustering coefficient is uniformly high. Additionally, it is important to remember that the triangles in these networks are association links and not necessarily sequences of plays. Hence we decided that the most meaningful measure to characterize the association structure of the ball movements was to calculate the clustering coefficients for undirected unweighted graphs across the different cutoffs of the cumulative weight, beginning with the 30 percentile when triangles first appear. This allowed us to compare teams with consistently high clustering to those that showed triangles only when less frequent links were included.

## Results and Discussion

The first question posed by this study was how well a network approach can capture the game of basketball from a team-level perspective. We constructed transition networks (i.e. stochastic matrices) as first-order characterization of team play style for each team individually and for the pooled set of all observed transitions across all teams. Because even a single game generates a rich dataset, we imposed thresholds to clarify the dominant transitions, highlighting from most to least frequent the minimal set of transitions representing a particular percentile of all ball movements. At the 60th percentile, players in all but one network were connected to at least one other player (the San Antonio Spurs Center was disconnected) and all teams had an edge to at least one outcome, generally success. This matched the expectation that these are elite and cohesive teams and gave us a starting point for comparative analyses (weighted graphs for all teams across the 30th to 70th percentile thresholds shown in Supplemental Figures S1 and S2).

**Figure 1:**
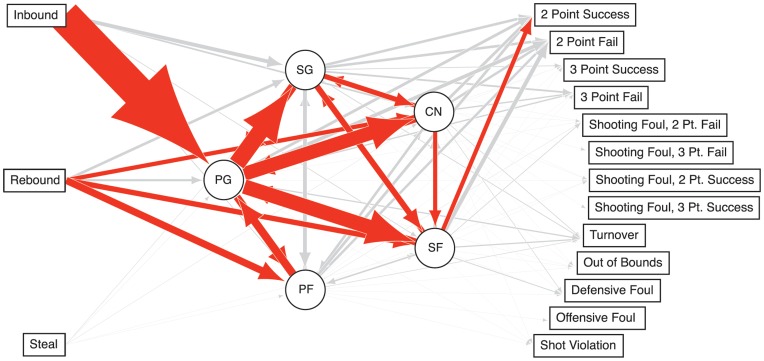
Weighted graph of ball transitions across all teams and all games. Edge width is proportional to probability of transition between nodes. Red edges represent transition probabilities summing to the 60^th^ percentile.

To look at the NBA as a whole, we combined the transition data across all teams in a compiled network (Figure 1). As a note, although it is tempting to relate the structure of play to physical location on the court, it is important to remember that these data capture passing probabilities independently of spatial information. In this network, as in an NBA game, the ball moved most frequently from the inbound pass to the Point Guard and was rebounded either by the Center or Power Forward. It was primarily distributed from the Point Guard to other players, with most likely distributions to the Shooting Guard or Power Forward. Other players generally distributed back to the Point Guard, with lower weights to edges connecting the Shooting Guard, Power Forward and Small Forward. The only edge to an outcome at this weighting was from the Power Forward to a successful shot. This NBA team thus showed a star-shaped pattern of ball movement controlled centrally by the Point Guard, with a division of labor across positional roles. Transitions from other players were most likely to be towards the Point Guard. The Shooting Guard occupied a secondary leadership role by creating connections between the Point Guard and the Power Forward who functioned as the primary shot-taker. The role of the Center was rebounding and redistribution to the Point Guard.

**Figure 2:**
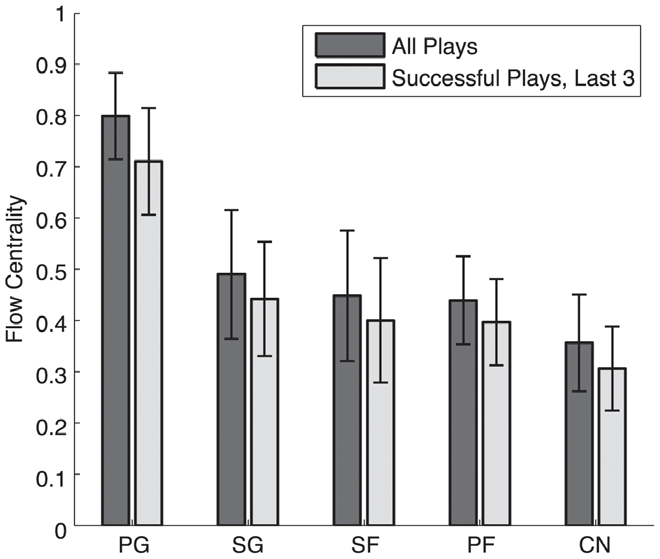
Mean flow centrality by position (+/− S.D.). Dark bars represent flow centrality calculated across all player possessions in a sequence, and light bars represent flow centrality calculated across the last 3 player possessions in successful sequences.

The importance of the Point Guard in distributing the ball identifies this as the primary leadership position in the team network. If we define leadership as the relative importance of any player or position in the network, we can capture this quantitatively using individual flow centrality, or the proportion of paths (offensive plays) involving a particular node [16]. We compared flow centrality across positions from all data (ANOVA; F = 42.02; P = 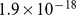; df = 4, n = 80 (Table S2); and for the three players contacting the ball before a shot (F = 36.12; P = 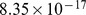). As expected from the network graphs, the Point Guard position had the highest mean centrality across all positions and was highest for the majority of teams (Figure 2). Flow centrality was conversely lowest for the Center, with intermediate and similar values for other positions. Two notable (but unsurprising) exceptions to this rule were the Cleveland Cavaliers, for which the Small Forward had high flow centrality, and the Los Angeles Lakers, for which the flow centrality of the Shooting Guard matched that of the Point Guard. These deviations match leadership roles within these teams by LeBron James and Kobe Bryant respectively. It will be interesting to compare their shifting network roles as their teams have changed; one moved to a team with an increased number of skilled offensive players (and the winning team in 2012), and the other’s team recently gained a new point guard (Steve Nash) known as an offensive strategist.

### Team Network Graphs

**Figure 3:**
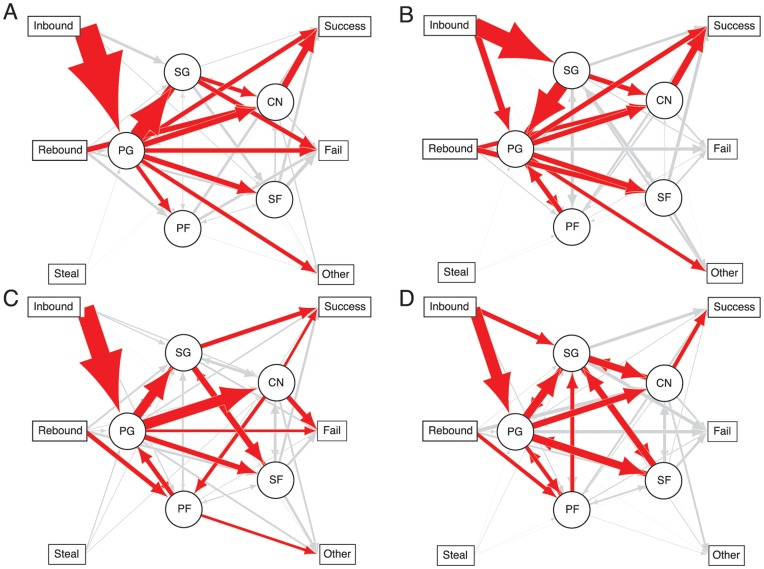
Weighted graphs of ball transitions across two games for the (a) Bulls, (b) Cavaliers, (c) Celtics and (d) Lakers. Red edges represent transition probabilities summing to the 60^th^ percentile. Player nodes are sorted by decreasing degree clockwise from the left.

How do individual teams vary around this centralized model? The star pattern was most exemplified by the Bulls (Figure 3), who inbound only to the Point Guard at 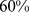, and for which most passes were between the Point Guard and other players. Their high degree centrality is illustrated by considering that removing the point guard node would cause all other player nodes to be completely disconnected. A similar disconnect would happen to five of the sixteen teams at 60% weighting and nine teams at 50% weighting (Figure S1 and S2). There are trade-offs to a highly centralized team between clarity of roles and flexibility of response. Lack of player connectedness may allow the defense to exploit a predictable weakness in the network by moving defenders off disconnected players to double team.

**Figure 4:**
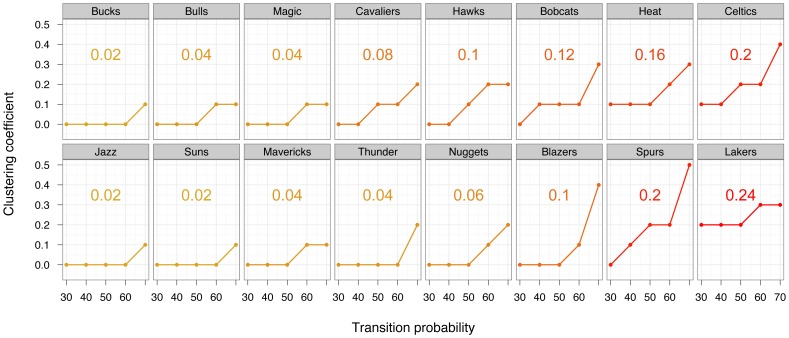
Clustering coefficients for the graphs of each team for cumulative transition probabilities between 30% and 70% of all ball movements. Networks are ordered according to the average clustering coefficient across all cutoffs.

Deviations from the Point-Guard centered star pattern confirmed known team playing styles (Figure 3). In the 2010 Cleveland Cavaliers network the Small Forward was a highly weighted distributor of the ball, as expected by his high flow centrality (Figure 2). He also shot the ball successfully at an edge weight close to the Power Forward. Thus the network visualization again picked up Le Bron James combined skills in ball distribution and shooting. However, perhaps the most important deviation from a centralized network strategy appeared in the weighted graphs of the Los Angeles Lakers. Even at low weighting, their network included multiple between-player edges beyond those connecting to the Point Guard. One way to analyze the impact of these additional edges is by quantifying the frequency of triangles within the network [17] via a clustering coefficient [14]. Figure 4 shows the cumulative clustering coefficients of each team from the 30th to 70th percentile weighting. The Lakers had the highest cumulative clustering coefficient, primarily because they had high connectedness in their most frequent plays. In a highly clustered network like the Lakers, passing decisions are made by multiple players, expanding the possible paths that must be considered by the opposing team. In the 2010 first round only two other teams showed comparable cumulative clustering: the Boston Celtics and the San Antonio Spurs. Like the Lakers, the Celtics - who also reached the finals - built triangles even at relatively low weighting. The Spurs were unusual in that they had low connectedness when considering their most dominant edges, but high clustering when less frequent passes were included in the analysis (i.e. at the 70th percentile).

The network concept of triangles as a fully connected subgroups translates well to the Lakers highly discussed triangle offense. Jackson and Winter [8] define the triangle offense as a spatial concept, in which a group of three players is set up on one side of the court connecting to a balanced two-man set on the other side. It is designed to distribute players across the floor so that they can be used interchangeably, depending on open lanes and defense. In this strategy the Point Guard becomes less central to the decision process, because all players have the ability to make decisions about ball distribution depending on immediate context. Thus the triangle offense can be considered as a network strategy that can be visualized in the Lakers weighted graph.

### Team Network Signatures: Degree Centrality and Entropy

An important question is whether differences in the weighted team graphs can be captured more quantitatively by network metrics. As discussed above, a primary visual distinction in our weighted graphs was between teams using a central player to distribute the ball, and those moving the ball across multiple players. Our calculated degree centralities in general matched our visual networks (Table 2). The data were not definitive, however, in whether less centralized teams had an advantage in the 2010 play-offs. Five of the 8 winning teams had lower degree centralities than opponents, but overall rankings of centrality showed no pattern of win/loss.

**Table 2:** Degree centrality, team entropy, and uphill/downhill flux measured across two games for the 16 teams in the 2010 playoffs.

		Degree Centrality		Team Entropy		Uphill/Downhill
1	Lakers*	0.084	Lakers*	3.234	Mavericks	0.093
2	Spurs*	0.087	Celtics*	3.229	Jazz*	0.044
3	Heat	0.089	Bobcats	3.224	Nuggets	0.025
4	Bobcats	0.093	Heat	3.194	Lakers*	0.016
5	Celtics*	0.117	Nuggets	3.189	Bucks	0.009
6	Blazers	0.119	Hawks*	3.180	Blazers	0.007
7	Mavericks	0.127	Magic*	3.178	Bobcats	0.005
8	Bucks	0.135	Spurs*	3.171	Celtics*	0.001
9	Thunder	0.148	Suns*	3.132	Cavaliers*	0.001
10	Suns*	0.154	Thunder	3.119	Bulls	0.000
11	Cavaliers*	0.158	Blazers	3.117	Magic*	−0.001
12	Nuggets	0.162	Cavaliers*	3.112	Suns*	−0.001
13	Magic*	0.171	Bucks	3.079	Spurs*	−0.003
14	Hawks*	0.176	Bulls	3.041	Hawks*	−0.006
15	Jazz*	0.211	Mavericks	2.949	Heat	−0.014
16	Bulls	0.219	Jazz*	2.934	Thunder	−0.048

(*) indicates the winner of the series.

Like degree centrality, entropy should be strongly influenced by the extent to which multiple players distribute the ball. Degree centrality and team entropy were negatively correlated (Pearson product moment correlation = −0.6; p<0.003; n = 16), but they captured somewhat different aspects of ball distribution, because team entropy takes into account probabilities outside the network topology. Variation in team entropy was more closely connected to individual team success/failure; winners in 6 of the 8 first round match-ups had higher team entropy, and when entropies were ranked from highest to lowest, 5 of the 8 highest entropies were for winning teams. The play-offs only provide 8 match-ups, too small a sample size to make a statistically meaningful claim (and it would be a simplistic game that allowed a predictive single metric). However, our analyses do suggest that these combined network metrics have value in: (1) capturing variation in team offense, and (2) supporting the hypothesis that complex and unpredictable ball distribution pattern is an important component of team strategy. Indeed, the 2010 Lakers and Celtics teams were arguably built around this principle. The highest entropies overall were achieved by the Lakers and Celtics, and the Lakers simultaneously had the lowest degree centrality. These assertions would be tested by the subsequent play-off seasons, one in which a team known for its dominant forward was successful (2011 Dallas Mavericks) and the next in which the winning team was built around the multi-player model (2012 Miami Heat).

### Uphill-downhill Flux and Passing Rate

**Figure 5:**
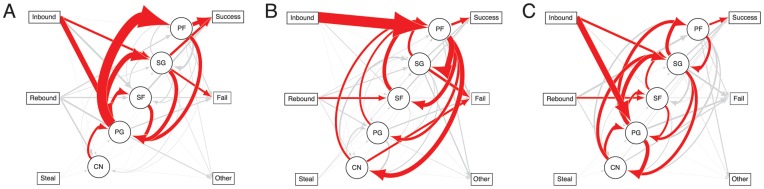
Weighted graphs of ball transitions with nodes sorted from lowest to highest scoring success illustrate uphill-downhill flux. Data collected across two games for the (a) Mavericks (highest uphill/downhill), (b) Thunder (lowest uphill/downhill), and (c) Lakers.

The Dallas Mavericks, who lost in the first round in 2010 but won the title in 2011, are an important counter-point. Their strategy was clear; move the ball consistently to their best shooter. To capture this quantitatively, we developed a new metric that uses flow flux to compare individual player flow centrality with calculated shooting percentage for each player across the two games. Uphill/downhill flux measures the degree to which teams move the ball towards versus away from players relative to their differential shooting success (Figure 5). High uphill/downhill indicates a different set of priorities in ball distribution than entropy. It focuses on playing to strengths by separating the roles of ball distribution and scoring, moving from distributors to shooters. Unsurprisingly, the 2010 Mavericks had the highest uphill/downhill flux of all teams in the play-offs. Success in this strategy was not connected consistently to team success within our data set. However, it is notable that only three teams had a combination of both higher uphill/downhill and higher entropy than their opponents. Two of the three were the Lakers and the Celtics; the third was the Heat.

Our final team-level metrics were path length and flow rate (speed of ball movement through the path; Table 3). Recently, there has been increased interest in small ball teams, which distribute the ball quickly across players. Small ball has been hypothesized to allow teams to achieve success beyond what would be expected based on individual player skill levels. The exemplar small ball team in past years has been the Phoenix Suns [18]. However, in 2009–2010 they transitioned away from this approach. We predicted a correlation between path length and flow rate, such that some teams distribute the ball quickly and across multiple players, but surprisingly little variation in path length or ball movement speed showed in our data.

**Table 3:** Path length and flow rate measured across two games for the 16 teams in the 2010 playoffs.

	Path Length		Flow Rate	
	Mean	Variance	Mean	Variance
Lakers*	5.81	3.67	0.60	0.28
Blazers	5.52	3.53	0.53	0.22
Heat	5.28	3.83	0.72	0.66
Mavericks	5.24	2.89	0.50	0.16
Bobcats	5.15	2.09	0.58	0.37
Spurs*	5.14	1.87	0.46	0.17
Bucks	4.96	1.94	0.55	0.34
Celtics*	4.93	2.75	0.68	0.52
Thunder	4.88	3.15	0.65	0.35
Nuggets	4.77	1.81	0.57	0.34
Cavaliers*	4.72	1.74	0.59	0.38
Jazz*	4.70	1.55	0.52	0.24
Hawks*	4.69	2.22	0.71	0.74
Suns*	4.68	1.88	0.53	0.22
Magic*	4.65	1.91	0.55	0.28
Bulls	4.48	1.62	0.69	0.53

(*) indicates the winner of the series.

### Player Value

A question in evaluating any organizational network is the relative value of its individual members [11]. Duch et al. [16] used individual flow centrality to show that higher paid players in soccer teams are in fact strong contributors to ball movement during a game. We asked a similar question for basketball, by quantifying player involvement in paths with successful versus unsuccessful outcomes. For our analyses we used only those sequences with at least 3 of the 5 starting players on the floor. We matched each player to position and excluded any sequences in which starters clearly rotated into a different position than assigned. This allowed us to analyze individual player contribution by position, using flow centrality analyses to determine the relative frequency by which any player was involved in (1) all, (2) only successful, and (3) only unsuccessful plays. We used the ratio of (2) to (3) to determine whether we could quantify player “value” beyond apparent dominance in the game (Table 4).

**Table 4:** Ratio of player flow centrality for successful versus unsuccessful plays.

Team	Position				
	PG	SG	SF	PF	CN
Bobcats	0.94	0.87	1.17	0.92	1.42
Bucks	0.94	0.65	1.25	0.87	1.54
Bulls	0.72	0.55	0.95	0.78	1.36
Cavaliers	0.76	1.24	0.81	1.51	0.87
Celtics	1.01	0.88	1.44	1.43	0.96
Hawks	0.95	1.00	0.54	0.76	0.82
Heat	0.54	1.63	0.97	0.78	0.48
Magic	1.07	0.55	0.94	0.91	1.70
Blazers	0.99	0.77	1.24	0.77	1.86
Jazz	0.95	1.13	0.86	1.09	0.80
Lakers	0.67	0.85	0.63	1.31	1.94
Mavericks	1.04	0.87	1.13	1.51	0.60
Nuggets	0.99	1.06	1.06	0.62	1.33
Spurs	1.04	1.00	1.83	0.58	1.70
Suns	0.96	0.76	1.20	1.29	0.34
Thunder	0.89	0.50	0.83	0.46	1.52

Flow centrality is calculated for the last three player possessions across plays.

We found an interesting positional bias in the data, with the Center often having the highest success/failure ratio. In contrast, Point Guards tended to have success/failure ratios at or below 1.0. Although the ratio measure should statistically control for frequency effects, we suggest this metric might be biased mechanistically by relative player involvement. The low flow centrality of the most highly utilized position reflects the argument that high frequency player contributions become negatively affected by exposure. The nonlinear relationship between player involvement and success in our metrics may thus illustrate the price of anarchy [13], the expectation that maximizing gain within any given offensive play can ultimately jeopardize overall game efficiency. If entropy is valuable, as our data suggest, then moving the ball frequently to a specific player or position is costly, because it allows the opposition to adjust their defense accordingly.

### Conclusion

We have presented a network structure analysis of basketball teams in the context of team coordination and strategy. As a starting point, we applied network-level metrics to quantitatively measure fundamental components of team offensive strategy, moving currently available individual player metrics (examples at NBA.com). The study involved more than a thousand ball movements and typically more than one hundred sequences or paths for each team. This dataset allowed us to capture the game of basketball as a network. Because our team comparisons were limited to the pairs in the first round of the play-offs, correlations between game outcome and specific aspects of network structure could not definitively test the specific hypotheses suggested. Answering the question of how network dynamics contribute to successful team strategy will be more complex than a single network variable can capture. We also expect intransitivity across games and opponents, such that the success of emphasizing any given strategy is dependent on the behavior of the opposing team. However our data do suggest that certain metric combinations, particularly entropy, centrality, and clustering, are relevant components of team strategy.

One of the advantages of this beautiful game is the wealth of available data. We encourage the expansion of both the network toolbox and the datasets analyzed. Analyses across a season will help determine whether network structures for a given team are stable or whether they respond flexibly to different defense strategies. Dissecting network shifts within games (e.g. the final quarter or as point differentials change) could help explore game dynamics. Analyses across multiple seasons could track the development of team cohesion. It would also be extremely useful to connect network with spatial and temporal models; this may not be practical with current data acquisition methods, but recent publications [19] suggest that automated ball tracking in basketball games is becoming more feasible.

Beyond basketball, this approach may act as a template for evaluating other small team collaborations. Although the specific network metrics will vary across the disparate contexts in which teams occur, the general approach of analyzing network interactions and function is robust [14]. Teams take multiple approaches to communication and leadership, from centralized to decentralized, from more rigidly bureaucratic to flexible, and from assigned roles to emergent. Each of these organizational strategies corresponds with a specific network model. As one example, our finding that the more successful teams distributed decision making about ball movement beyond a centralized leader is mirrored in models of business team structure. Network assessments suggest that business teams with mixed leadership roles optimize performance relative to highly centralized or highly distributed teams [6]. It would be interesting to see how the network measures used here apply to other small teams that are tasked differently, such as research groups organized around innovation, remote military teams on assignment, or intelligence agencies tasked with pattern recognition. The application could also be expanded to animal teams in which roles develop naturally rather than through external assignment, and for which team success/failure has a direct connection to fitness. For example, the ontogeny of team coordination is a general phenomenon. In hunting teams of lions, chimpanzees and wild dogs, new members can require years of practice to achieve coordination with the group [1]–[3]. These discussions highlight the potential of this approach and its applicability across the broad array of contexts in which cohesive teams are found.

## Supporting Information

Figure S1 **Weighted graphs of ball movement for East Coast teams.** Red edges represent transition probabilities summing to the percentile indicated in the column header.
(PDF)

Figure S2 **Weighted graphs of ball movement for all West Coast teams.** Red edges represent transition probabilities summing to the percentile indicated in the column header.
(PDF)

Table S1 **Starting players and position assignments for the 2010 NBA playoffs, first round.** Substitutes are in parentheses.
(PDF)

Table S2 **Player flow centrality**. Flow centrality (FC) is calculated as the proportion of all plays in which a player was involved. Flow centrality based on outcome is calculated as the proportion of successful (FC3 S) or failed (FC3 F) plays in which a player appears as one of the last 3 player possessions in the sequence.
(PDF)
