# Supplementary figures and images for: Basketball Teams as Strategic Networks
Source: PLoS One. 2012 Nov 6;7(11):e47445. doi: 10.1371/journal.pone.0047445 (PMC3490980; doi:10.1371/journal.pone.0047445)

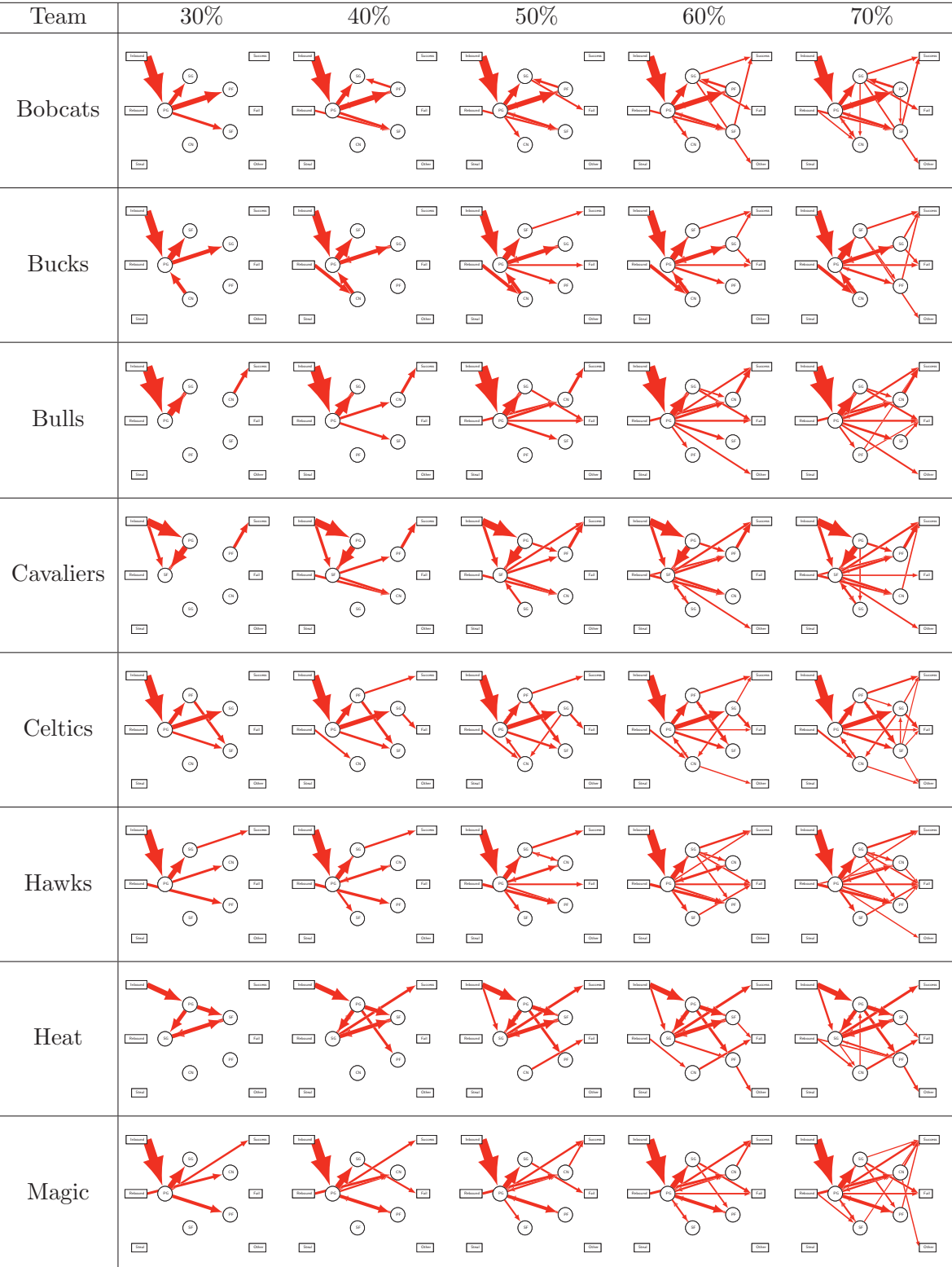

Supplement: Figure S1 — Weighted graphs of ball movement for East Coast teams. Red edges represent transition probabilities summing to the percentile indicated in the column header. (PDF) [file pone.0047445.s001.pdf]

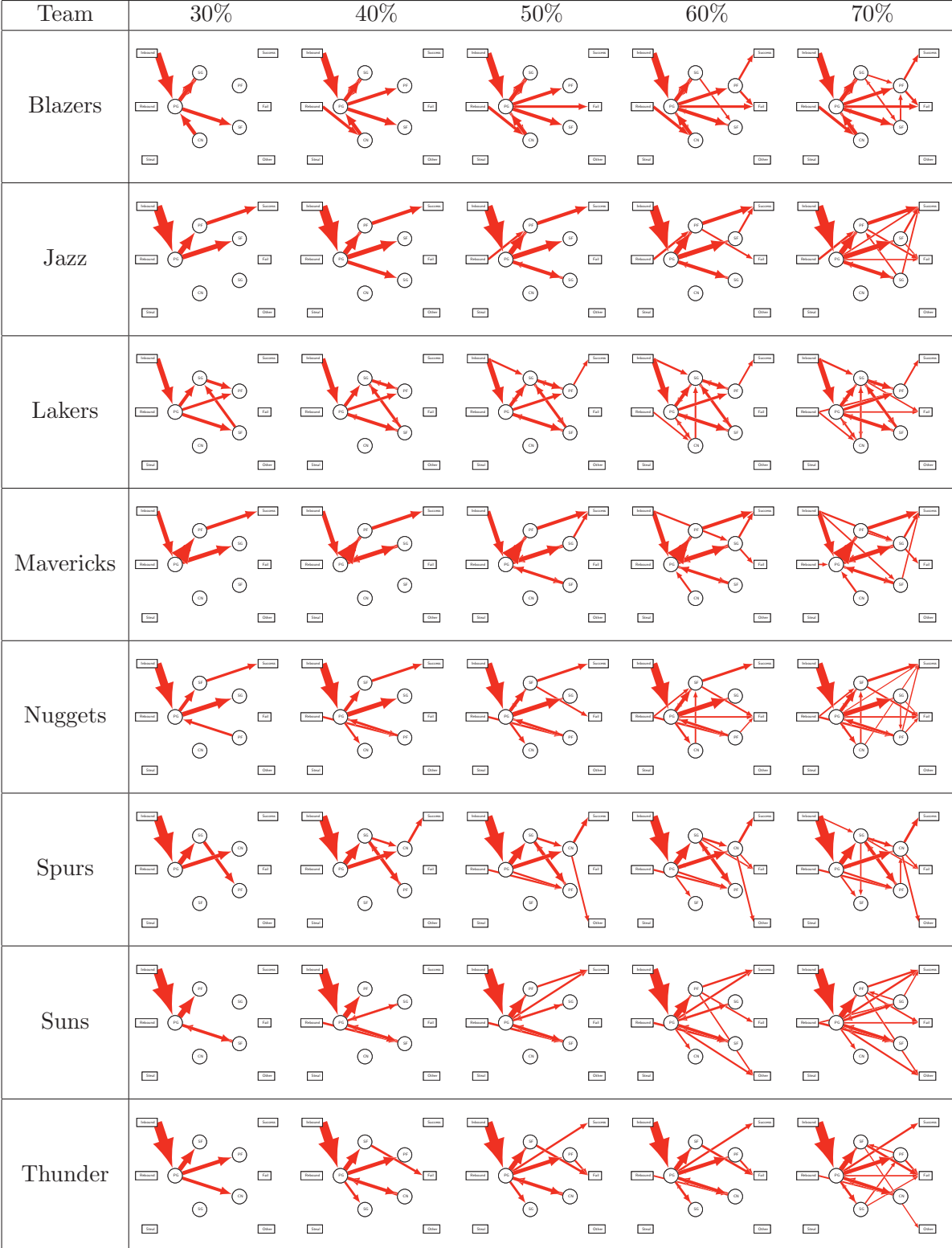

Supplement: Figure S2 — Weighted graphs of ball movement for all West Coast teams. Red edges represent transition probabilities summing to the percentile indicated in the column header. (PDF) [file pone.0047445.s002.pdf]
