# Supplementary material for: Basketball Teams as Strategic Networks
Source: PLoS One. 2012 Nov 6;7(11):e47445. doi: 10.1371/journal.pone.0047445 (PMC3490980; doi:10.1371/journal.pone.0047445)
Supplement: Table S1 — Starting players and position assignments for the 2010 NBA playoffs, first round. Substitutes are in parentheses. (PDF) [file pone.0047445.s003.pdf]

Table S1: Starting players and position assignments for the 2010 NBA playoffs, first round. Substitutes are in parentheses.

Team	Position 1<br>Point Guard	Position 2<br>Shooting Guard	Position 3<br>Small Forward	Position 4<br>Power Forward	Position 5<br>Center
Blazers	#24 Andre Miller<br>(#4 Jarryd Bayless)	#7 Brandon Roy<br>(#5 Rudy Fernandez)	#88 Nicolas Batum<br>(#23 Martell Webster)	#12 L. Aldridge<br>(#6 Juwan Howard)	#21 Marcus Camby<br>(#33 D. Cunningham)
Bobcats	#20 Ray Felton<br>(#14 D.J. Augustin)	#1 Stephen Jackson<br>(#0 Larry Hughes)	#3 Gerald Wallace	#32 Boris Diaw	#42 Theo Ratliff<br>(#13 N. Mohammed)
Bucks	#3 B. Jennings<br>(#13 Luke Ridnour)	#10 Carlos Delfino<br>(#24 J. Stackhouse)	#15 John Salmons	#12 L. Mbah a Moute<br>(#7 Ersan Ilyasova)	#40 Kurt Thomas<br>(#50 Dan Gadzuric)
Bulls	#1 Derrick Rose	#12 Kirk Hinrich<br>(#6 Ronald Murray)	#9 Luol Deng<br>(#16 James Johnson)	#52 Brad Miller<br>(#22 Taj Gibson)	#13 Joakim Noah
Cavaliers	#2 Mo Williams<br>(#13 Delonte West)	#18 Anthony Parker	#23 LeBron James	#4 Antawn Jamison<br>(#17 A. Varejao)	#33 Shaquille O'Neal<br>(#11 Z. Ilgauskas)
Celtics	#9 Rajon Rondo<br>(#4 Nate Robinson)	#20 Ray Allen<br>(#42 Tony Allen)	#34 Paul Pierce<br>(#40 Michael Finley)	#5 Kevin Garnett<br>(#11 Glen Davis)	#43 Kendrick Perkins
Hawks	#10 Mike Bibby<br>((#11 J. Crawford)	#2 Joe Johnson	#24 Marvin Williams<br>(#1 Maurice Evans)	#5 Josh Smith	#15 Al Horford<br>(#27 Zaza Pachulia)
Heat	#8 Carlos Arroyo	#3 Dwyane Wade<br>(#6 Mario Chalmers)	#5 Q. Richardson<br>(#1 Dorell Wright)	#30 Michael Beasley<br>(#40 Udonis Haslem)	#7 Jermaine O'Neal
Jazz	#8 Deron Williams	#23 W. Matthews<br>(#26 Kyle Korver)	#34 C.J. Miles	#5 Carlos Boozer	#24 Paul Millsap<br>(#44 K. Fesenko)
Lakers	#2 Derek Fisher<br>(#1 Jordan Farmar)	#24 Kobe Bryant	#37 Ron Artest	#16 Pau Gasol	#17 Andrew Bynum<br>(#7 Lamar Odom)
Magic	#14 Jameer Nelson<br>(#44 J. Williams)	#15 Vince Carter<br>(#7 J.J. Redick)	#22 Matt Barnes<br>(#20 Mickael Pietrus)	#9 Rashard Lewis<br>(#33 Ryan Anderson)	#12 Dwight Howard<br>(#13 Marcin Gortat)
Mavericks	#2 Jason Kidd<br>(#11 Jose Barea)	#4 Caron Butler<br>(#31 Jason Terry)	#0 Shawn Marion<br>(#3 R. Beaubois)	#41 Dirk Nowitzki	#25 Erick Dampier<br>(#33 B. Haywood)
Nuggets	#1 C. Billups	#6 Arron Afflalo<br>(#5 J.R. Smith)	#15 Carmelo Anthony	#4 Kenyon Martin	#31 Nene Hilario<br>(#11 C. Andersen)
Spurs	#3 George Hill<br>(#9 Tony Parker)	#20 Manu Ginobili	#24 Richard Jefferson	#34 A. McDyess<br>(#15 Matt Bonner)	#21 Tim Duncan
Suns	#13 Steve Nash<br>(#2 Goran Dragic)	#23 J. Richardson	#33 Grant Hill<br>(#3 Jared Dudley)	#1 A. Stoudemire<br>(#17 L. Amundson)	#20 Jarron Collins<br>(#8 Channing Frye)
Thunder	#0 R. Westbrook<br>(#6 Eric Maynor)	#2 Thabo Sefolosha<br>(#13 James Harden)	#35 Kevin Durant	#22 Jeff Green	#12 Nenad Krstic<br>(#4 Nick Collison)
