# Supplementary material for: Basketball Teams as Strategic Networks
Source: PLoS One. 2012 Nov 6;7(11):e47445. doi: 10.1371/journal.pone.0047445 (PMC3490980; doi:10.1371/journal.pone.0047445)
Supplement: Table S2 — Player flow centrality. Flow centrality (FC) is calculated as the proportion of all plays in which a player was involved. Flow centrality based on outcome is calculated as the proportion of successful (FC3 S) or failed (FC3 F) plays in which a player appears as one of the last 3 player possessions in the sequence. (PDF) [file pone.0047445.s004.pdf]

Table S2: Player flow centrality. Flow centrality (FC) is calculated as the proportion of all plays in which a player was involved. Flow centrality based on outcome is calculated as the proportion of successful (FC3 S) or failed (FC3 F) plays in which a player appears as one of the last 3 player possessions in the sequence.

Team	FC					FC3 S					FC3 F				
	PG	SG	SF	PF	CN	PG	SG	SF	PF	CN	PG	SG	SF	PF	CN
Bobcats	0.86	0.57	0.45	0.47	0.38	0.67	0.53	0.38	0.40	0.40	0.72	0.61	0.33	0.43	0.28
Bucks	0.87	0.43	0.50	0.39	0.35	0.81	0.33	0.54	0.35	0.38	0.86	0.51	0.43	0.41	0.24
Bulls	0.79	0.47	0.30	0.23	0.40	0.61	0.36	0.27	0.20	0.45	0.86	0.67	0.29	0.26	0.33
Cavs	0.66	0.28	0.79	0.39	0.37	0.57	0.31	0.69	0.45	0.26	0.75	0.25	0.85	0.30	0.30
Celtics	0.82	0.48	0.44	0.49	0.36	0.66	0.43	0.43	0.54	0.29	0.65	0.49	0.30	0.38	0.30
Hawks	0.70	0.57	0.29	0.37	0.39	0.63	0.61	0.16	0.32	0.29	0.67	0.60	0.29	0.42	0.35
Heat	0.66	0.68	0.57	0.43	0.34	0.34	0.76	0.52	0.21	0.21	0.63	0.47	0.53	0.27	0.43
Magic	0.84	0.48	0.32	0.38	0.37	0.78	0.33	0.27	0.33	0.37	0.73	0.61	0.29	0.37	0.22
Blazers	0.90	0.52	0.44	0.49	0.47	0.82	0.39	0.42	0.39	0.42	0.83	0.51	0.34	0.51	0.23
Jazz	0.91	0.36	0.43	0.52	0.12	0.82	0.36	0.43	0.55	0.11	0.86	0.32	0.50	0.50	0.14
Lakers	0.70	0.72	0.56	0.53	0.47	0.40	0.55	0.35	0.53	0.48	0.60	0.64	0.56	0.40	0.24
Mavs	0.83	0.51	0.40	0.60	0.23	0.77	0.48	0.36	0.68	0.14	0.74	0.55	0.32	0.45	0.23
Nuggets	0.74	0.41	0.50	0.43	0.35	0.73	0.36	0.50	0.30	0.39	0.74	0.34	0.47	0.47	0.29
Spurs	0.79	0.65	0.30	0.44	0.48	0.70	0.48	0.35	0.30	0.57	0.67	0.48	0.19	0.52	0.33
Suns	0.82	0.38	0.39	0.49	0.23	0.69	0.36	0.33	0.54	0.10	0.72	0.47	0.28	0.42	0.31
Thunder	0.90	0.33	0.49	0.38	0.39	0.77	0.15	0.35	0.23	0.42	0.86	0.31	0.42	0.50	0.28
